# Topics in Accelerator Health Physics: HPS Professional Development School Proceedings

**Published:** 2009

**Authors:** Marc Mackenzie

**Affiliations:** Department of Medical Physics, Cross Cancer Institute, Edmonton, Alberta, Canada. E-mail: marcmack@cancerboard.ab.ca

“Topics in Accelerator Health Physics” is a recently published compendium from the U.S. Health Physics Society (USHPS) of material originally presented at their Professional Development School held in January, 2008, at Oakland, CA. The presenters were current or retired professionals from various national labs or university based research accelerators (TRIUMF, SLAC, Los Alamos, Argonne, Fermi, Loma Linda, Thomas Jefferson, and Pacific NW).

After an initial general chapter on accelerator physics for health physicists, other chapters covered some pertinent fundamental radiation physics, practical topics and professional/ administrative topics. The general accelerator physics of interest to health physicists included topics such as mechanisms of - prompt and induced radioactivity, radiation damage to materials. Practical topics covered include topics such as monitoring (area, personnel and environmental), shielding/shielding codes, and new accelerator facility design. Professional or administrative topics covered included general safety systems and health physics program administration, as well as regulatory issues. There are also general overview chapters describing synchrotron facilities, free electron lasers, accelerators specifically for radiation therapy, as well as new and emerging technologies.

While the topics covered will be of interest to health physicists working at both research and industrial facilities, some of this collection is of a slightly more academic bent than what the more clinically oriented health physicists are likely to encounter in their routine work. The academic tone of some chapters may, however, make it ideal for health physics professionals who are trying to brush up on their fundamentals.

The more technical chapters do not shy away from either the appropriate formulae or graphs; these chapters are followed by chapters that are more administrative in tone, describing radiation safety programs, monitoring and response to incidents. The case studies in the chapter on Health Physics Program Management were particularly interesting. Some chapters are extensive, perhaps a little excessively so, whereas other (Environmental Monitoring, for example) are given short shrift. However, on balance, this collection appears to provide a good overview of topics of interest to health physicists.

One issue from a Canadian perspective is that being a publication from the USHPS, the emphasis is naturally on US regulations and regulatory bodies, and there is some attention to “homeland security” issues. Nevertheless, general principles will still be universally applicable.

Although its breadth of coverage of different accelerators, and depth coverage of underlying physics may exceed what is required by many health physicists on a day-to-day basis, it can nevertheless serve as a good broad reference for members of the larger medical physics community especially if one is interested in topics not often covered elsewhere such as prompt activation or radiation damage to materials. If one is looking for a reference text for accelerator theory, however, there are better texts available.

In summary, this collection is quite likely an ideal reference for the health physicist working in a mixed administrative/research position in the United States, and a reasonably good reference for any medical physicist with radiation safety duties. It may still be of interest to other medical physicists, but there may be other reference texts that would serve them better, depending on their specific interests.

